# Supplementary figures and images for: The extant immunoglobulin superfamily, member 1 gene results from an ancestral gene duplication in eutherian mammals
Source: PLoS One. 2022 Jun 2;17(6):e0267744. doi: 10.1371/journal.pone.0267744 (PMC9162367; doi:10.1371/journal.pone.0267744)

Figure 6

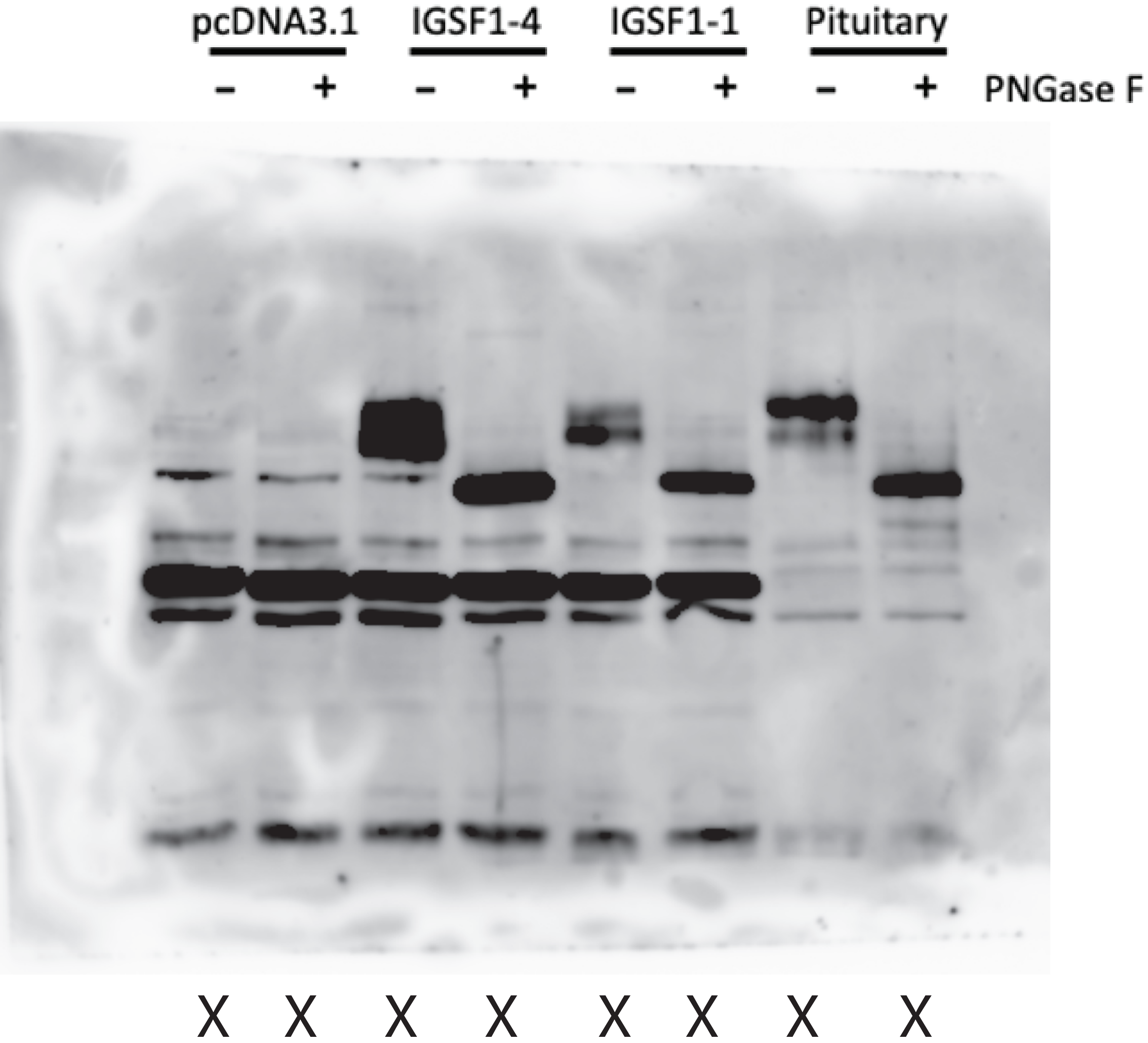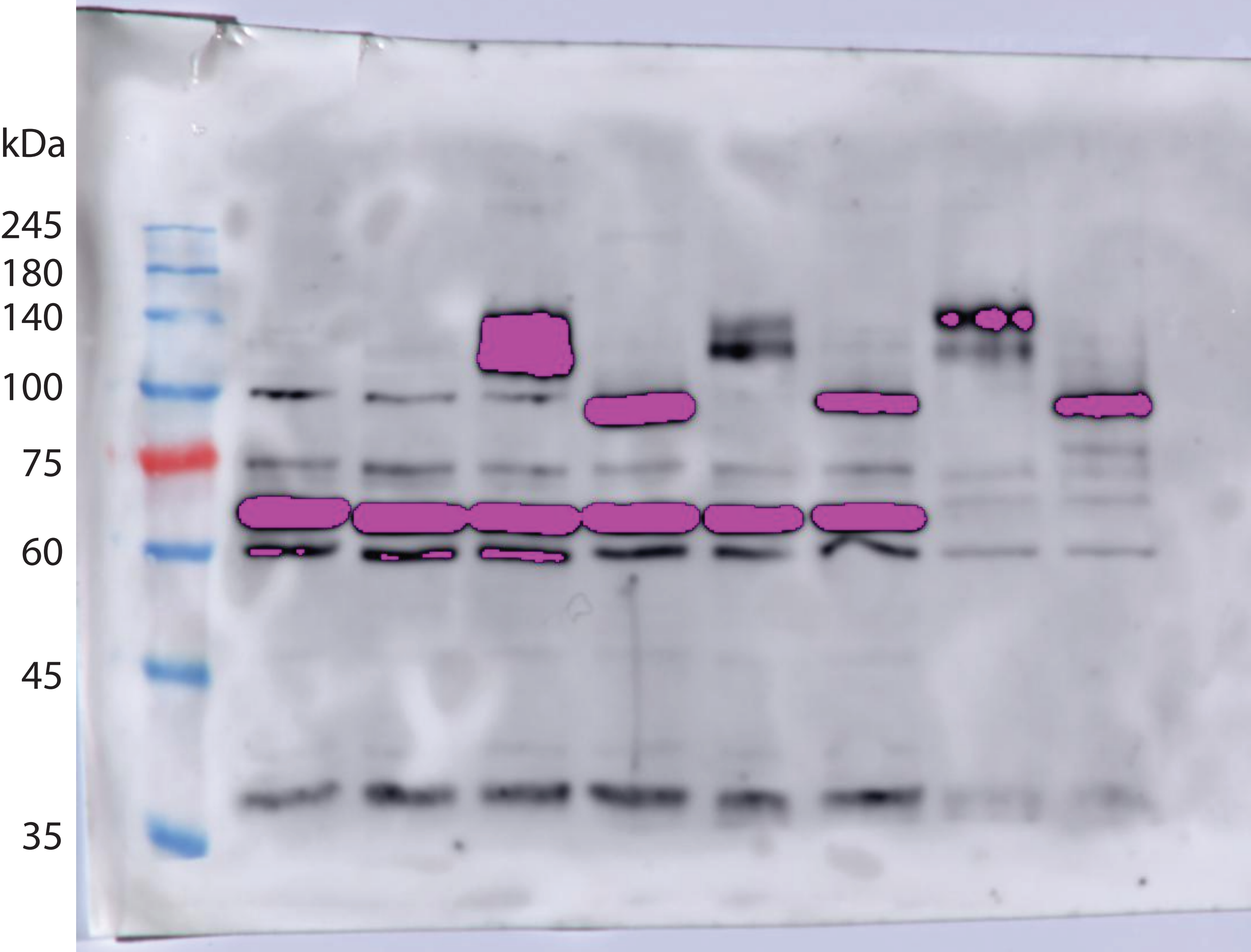

GE A1600 RBG chemiluminescence

IB: IGSF1-CTD

Supplement: S1 Raw images — (PDF) [file pone.0267744.s001.pdf]
